# Exploring virulence and immunogenicity in the emerging pathogen *Sporothrix brasiliensis*

**DOI:** 10.1371/journal.pntd.0005903

**Published:** 2017-08-30

**Authors:** Paula Portella Della Terra, Anderson Messias Rodrigues, Geisa Ferreira Fernandes, Angela Satie Nishikaku, Eva Burger, Zoilo Pires de Camargo

**Affiliations:** 1 Department of Medicine, Discipline of Infectious Diseases, Federal University of São Paulo (UNIFESP), São Paulo, Brazil; 2 Department of Microbiology, Immunology and Parasitology, Discipline of Cellular Biology, Federal University of São Paulo (UNIFESP), São Paulo, Brazil; 3 Department of Microbiology and Immunology, Federal University of Alfenas (UNIFAL-MG), Alfenas, Brazil; Rutgers University, UNITED STATES

## Abstract

Sporotrichosis is a polymorphic chronic infection of humans and animals classically acquired after traumatic inoculation with soil and plant material contaminated with *Sporothrix* spp. propagules. An alternative and successful route of transmission is bites and scratches from diseased cats, through which *Sporothrix* yeasts are inoculated into mammalian tissue. The development of a murine model of subcutaneous sporotrichosis mimicking the alternative route of transmission is essential to understanding disease pathogenesis and the development of novel therapeutic strategies. To explore the impact of horizontal transmission in animals (e.g., cat-cat) and zoonotic transmission on *Sporothrix* fitness, the left hind footpads of BALB/c mice were inoculated with 5×10^6^ yeasts (n = 11 *S*. *brasiliensis*, n = 2 *S*. *schenckii*, or n = 1 *S*. *globosa*). Twenty days post-infection, our model reproduced both the pathophysiology and symptomology of sporotrichosis with suppurating subcutaneous nodules that progressed proximally along lymphatic channels. Across the main pathogenic members of the *S*. *schenckii* clade, *S*. *brasiliensis* was usually more virulent than *S*. *schenckii* and *S*. *globosa*. However, the virulence in *S*. *brasiliensis* was strain-dependent, and we demonstrated that highly virulent isolates disseminate from the left hind footpad to the liver, spleen, kidneys, lungs, heart, and brain of infected animals, inducing significant and chronic weight loss (losing up to 15% of their body weight). The weight loss correlated with host death between 2 and 16 weeks post-infection. Histopathological features included necrosis, suppurative inflammation, and polymorphonuclear and mononuclear inflammatory infiltrates. Immunoblot using specific antisera and homologous exoantigen investigated the humoral response. Antigenic profiles were isolate-specific, supporting the hypothesis that different *Sporothrix* species can elicit a heterogeneous humoral response over time, but cross reaction was observed between *S*. *brasiliensis* and *S*. *schenckii* proteomes. Despite great diversity in the immunoblot profiles, antibodies were mainly derived against 3-carboxymuconate cyclase, a glycoprotein oscillating between 60 and 70 kDa (gp60-gp70) and a 100-kDa molecule in nearly 100% of the assays. Thus, our data broaden the current view of virulence and immunogenicity in the *Sporothrix*-sporotrichosis system, substantially expanding the possibilities for comparative genomic with isolates bearing divergent virulence traits and helping uncover the molecular mechanisms and evolutionary pressures underpinning the emergence of *Sporothrix* virulence.

## Author summary

Sporotrichosis is polymorphic infection that is acquired after traumatic implantation of *Sporothrix* propagules into host tissues. In Brazil, large epizooties occur in domestic cats and consequently massive zoonotic transmission has been observed since the 2000s without signs of decreasing over time. In this study, we explored a subcutaneous murine model of sporotrichosis mimicking the alternative route of transmission considering animal horizontal transmission (cat-cat) and zoonotic transmission (cat-human) in which *S*. *brasiliensis* yeasts are inoculated. Surprisingly, we found that virulence in the clonal offshoot *S*. *brasiliensis* is strain-dependent where highly virulent isolates can disseminate from the site of inoculation (footpad) to vital organs of infected animals, inducing dramatic weight loss, which correlates with host death. Such variation may be regarded as a function of the host's immune response delivered against *Sporothrix*. Additionally, humoral immune responses produced IgG against isoforms of 3-carboxymuconate cyclase (gp60-gp70) and a 100-kDa molecule. Our study demonstrated that increased or attenuated virulence occurs in *S*. *brasiliensis* considering the route of infection, morphotype inoculated and the mammalian immune response. In addition to improving our understanding of the pathogenesis of the emerging animal-born *S*. *brasiliensis*, this study has key implications for future developments in serological diagnosis and vaccination efforts.

## Introduction

Recent years have seen a global burden of fungal infections in warm-blooded hosts, many of which have the potential to trigger epidemics or cause endemic diseases [1]. Sporotrichosis is a chronic infection of humans and animals caused by *Sporothrix* species in the order Ophiostomatales, which is mainly composed of saprophytic fungi. Remarkably, a pathogenic group consisting of four species (*Sporothrix brasiliensis*, *Sporothrix schenckii*, *Sporothrix globosa*, and *Sporothrix luriei*) has emerged as an important threat to the health of several warm-blooded hosts around the globe [2]. As the genetic distance increases from this pathogenic group, basal phylogenetic groups of *Sporothrix* are usually associated with a decrease in infectivity for mammals. Therefore, rare agents of sporotrichosis are found outside the clinical group, such as *Sporothrix chilensis*, *Sporothrix mexicana*, *Sporothrix pallida* and *Sporothrix stenoceras* [3–7].

Among the clinical agents, *S*. *brasiliensis* is by far the most virulent, followed by *S*. *schenckii* and *S*. *globosa* [8, 9], but all of them are capable of disease in mostly healthy patients [10]. A myriad of virulence factors, such as thermotolerance, adherence factors, and melanin produced during the host-pathogen interplay, help *S*. *brasiliensis* invade mammalian hosts, cause sporotrichosis, and evade host defenses. In one study of experimental sporotrichosis, Arrillaga-Moncrieff *et al*. [9] employed a disseminated model of infection to reveal striking differences related to the species, source, and genetic background, suggesting that the pathogenesis of sporotrichosis could be species-specific. Similarly, Fernandes *et al*. [8] confirmed differential virulence in the *S*. *schenckii* clade (pathogenic clade) and proposed a correlation among protein secretion, immunogenicity, genetic diversity, and virulence.

Despite great efforts in understanding the pathogenesis of sporotrichosis in light of recent taxonomic changes in *Sporothrix* [8, 9, 11–13], only a few studies have used a subcutaneous model of infection [14–17]. This subcutaneous route of inoculation reflects the natural route of transmission of *Sporothrix* spp. and holds true for traumatic inoculation with soil and plant material contaminated with *Sporothrix* propagules or animal-driven inoculation [3]. A major difference between these sources of contamination is related to the morphotype inoculated; the environmental inoculum is expected to involve hypha and conidia [18], whereas animal-driven inoculation is expected to involve deep inoculation of yeasts into the subcutaneous tissue [19].

Hitherto poorly explored animal-driven inoculation deserve consideration for future developments in the system *S*. *brasiliensis*-sporotrichosis. Brito *et al*. [14] experimentally infected BALB/c mice by inoculating two strains of *S*. *schenckii* (*sensu lato*) subcutaneously into the left hind footpad. After challenge, animals presented with weight loss, cutaneous lesions, signs of inactivity, and different survival rates, mimicking human disease. Histological analysis revealed lesions in the organs, with inflammatory infiltrate and granuloma in the liver and inflammatory reaction in the area where inoculated.

In recent years, human and feline sporotrichosis due to *S*. *brasiliensis* has emerged as a major health problem in Brazil, initially affecting the metropolitan region of Rio de Janeiro, but quickly spreading to several urbanized areas, some as far as 2,000 km from the epicenter of the initial epidemics [20–22]. In the present study, we developed a subcutaneous murine model of infection mimicking the alternative route of transmission that includes both animal horizontal transmission (e.g., cat-cat) and zoonotic transmission (e.g., cat-human) in which a high load of *S*. *brasiliensis* yeast cells may be inoculated through feline bites and scratches. Afterwards, we explored this system to compare virulence levels among 11 isolates of *S*. *brasiliensis* and allied species based on the fungal tissue burden, survival assay, histopathological aspects of affected organs, weight loss, protein secretion, immunogenicity, and genetic diversity.

## Methods

### Ethical approval

The study was performed in strict accordance with recommendations in the Guide for the Care and Use of Laboratory Animals of the National Institutes of Health and approved by the Institutional Ethics in Research Committee of the Federal University of São Paulo (protocol number 8123190914).

### *Sporothrix* isolates

*Sporothrix* isolates were obtained from the Federal University of São Paulo (UNIFESP), São Paulo, Brazil. These isolates were characterized previously at the species level by phylogenetic analysis of the calmodulin-encoding gene [8, 23], species-specific PCR [24], and rolling circle amplification (RCA) [25]. We selected a set of *S*. *brasiliensis* (n = 11), *S*. *schenckii* (n = 2), and *S*. *globosa* (n = 1) based on the following criteria: clinical origins (human and feline), geographic region, idiomorphic mating type (*MAT1-1* and *MAT1-2*), and genetic diversity (Table 1). It is important to emphasize that although *S*. *brasiliensis* presents with little diversity during outbreaks [26, 27] our efforts were focused to choose the most genetically deviating ones. Reference strains were included in all experiments. The Ss06 (*S*. *globosa*), Ss39 (*S*. *schenckii*), Ss126 (*S*. *schenckii*), and Ss54 (*S*. *brasiliensis*) isolates were used as controls for low, medium, and high virulence levels as described previously [8]. Attenuation of virulence may occur more rapidly in some strains than others when subjected to successive *in vitro* subculturing and therefore impact multiple comparisons as proposed here. To prevent any bias among *Sporothrix* spp. isolates at the start of *in vitro* culturing, all isolates were passed through BALB/c and then re-isolated as a monosporic culture prior to challenge experiments [8].

**Table 1:** Characteristics of *Sporothrix* spp. isolates.

Isolate	Other code	Species^1^	Host	Origin^2^	Mating type^3^	Genbank^4^	Reference
**Ss34**	-	*S*. *brasiliensis*	Human	PR, Brazil	*MAT 1–1*	KF943638	[27]
**Ss54**	CBS 132990	*S*. *brasiliensis*	Feline	RS, Brazil	*MAT 1–1*	JQ041903	[8]
**Ss66**	-	*S*. *brasiliensis*	Human	RJ, Brazil	*MAT 1–2*	KF943649	[27]
**Ss67**	-	*S*. *brasiliensis*	Human	RJ, Brazil	*MAT 1–2*	KF943650	[27]
**Ss99**	-	*S*. *brasiliensis*	Human	RJ, Brazil	*MAT 1–2*	KF574460	[27]
**Ss104**	-	*S*. *brasiliensis*	Human	MT, Brazil	*MAT 1–2*	KF574461	[27]
**Ss174**	CBS 133002	*S*. *brasiliensis*	Feline	PR, Brazil	*MAT 1–1*	KC693874	[27]
**Ss226**	CBS 133003	*S*. *brasiliensis*	Feline	SP, Brazil	*MAT 1–2*	KC693875	[26]
**Ss252**	CBS 133011	*S*. *brasiliensis*	Feline	RJ, Brazil	*MAT 1–2*	KC693885	[26]
**Ss261**	-	*S*. *brasiliensis*	Human	RS, Brazil	*MAT 1–1*	KC693894	[26]
**Ss265**	CBS 133020	*S*. *brasiliensis*	Human	MG, Brazil	*MAT 1–1*	JN204360	[28]
**Ss06**	CBS 132922	*S*. *globosa*	Human	MG, Brazil	*MAT 1–1*	JF811336	[8]
**Ss39**	-	*S*. *schenckii*	Human	PR, Brazil	*MAT 1–2*	JQ041899	[8]
**Ss126**	-	*S*. *schenckii*	Human	SP, Brazil	*MAT 1–2*	JQ041904	[8]

^1^*Sporothrix* isolates were previously characterized at the species level by phylogenetic analysis of the calmodulin gene, species-specific PCR, and rolling circle amplification (RCA).

^2^PR, Paraná; RS, Rio Grande do Sul; RJ, Rio de Janeiro; MT, Mato Grosso; SP, São Paulo; MG, Minas Gerais.

^3^Mating type loci determined by PCR as described previously [29].

^4^Genbank accession codes for the calmodulin-encoding loci.

### Preparation of *Sporothrix* yeast cells for experimental infection

*Sporothrix* isolates were maintained in Sabouraud dextrose agar slants (Difco, Detroit, USA) and cultivated for 7 days at 25°C prior to use. Approximately 2×10^6^ conidia (90% viable cells) were used to inoculate 500-ml flasks containing 150 ml of Brain Heart Infusion Broth (Difco, Detroit, USA). The cultures were incubated at 37°C in a rotary shaker (Multitron II—Infors HT, Switzerland) with constant orbital agitation (100 rpm) for 4 days.

Yeast cells were collected by centrifugation at 3,500 g for 15 min (4°C) and then washed three times in phosphate buffered saline (PBS). The yeast cell concentration was adjusted to 5×10^6^ cells (25 μl) and cell viability assessed by trypan blue staining and by plating dilutions of the suspension on BHI plates as described previously [8, 30]. Only samples with ≥85% viability were employed for experimental infection.

### Animals

Male BALB/c mice weighing 25–30 g (6 to 8 weeks old) were obtained from Federal University of São Paulo (UNIFESP) for the virulence assays; 90 mice were used for colony-forming unit (CFU) assays and 150 for survival assays. Animals were housed in temperature-controlled rooms at 23–25°C, five per cage, in standard boxes with *ad libitum* access to food and water [8].

### Survival assay and weight loss

Animals were divided into 15 groups of 10 mice each and inoculated subcutaneously into the left hind footpad (one group for each *Sporothrix* isolate and one negative control group). The mice were anesthetized with 0.2 mg/kg xylazine and 20 mg/kg ketamine and subcutaneously inoculated with 25 μl containing 5×10^6^ yeast cells/animal. The control group received 25 μl of PBS only. The infected mice were observed daily and their mortality recorded over the following 16 weeks [8]. The percent weight loss was determined by measuring each animal’s weight every week post-inoculation (up to 16 weeks) and comparing it to the animal’s weight on the day of inoculation.

### CFU analysis and sera collection

Animals were divided into 15 groups of 6 mice each (one group for each *Sporothrix* isolate and one negative control group). The mice were anesthetized with 0.2 mg/kg xylazine and 20 mg/kg ketamine and subcutaneously inoculated with 5×10^6^ yeast cells/animal. The control group received 25 μl of PBS only. Twenty days post-infection, the animals were sacrificed by CO_2_ anesthesia and the liver, spleen, kidneys, lungs, heart, brain, and footpad aseptically removed. The organs and footpad were separated, weighed, and homogenized in sterile PBS using a tissue grinder. Samples (100 μl) of each homogenate were seeded on Petri dishes containing BHI agar and incubated at 37°C. Colonies were counted from day 15 to 20. The results were expressed as CFU/g tissue [30]. Serum was collected from the mice and stored at –20°C for Western blot as described elsewhere [8].

### Statistical analysis

CFU assay results were compared among groups and analyzed by analysis of variance (ANOVA) followed by post-hoc Tukey. Significance was set at *P*≤0.05. For survival assays, data were analyzed by Kaplan-Meier survival plots followed by the log-rank test. For weight loss assays, data were analyzed by paired t-test. *P*≤0.05 was considered significant. All analyses were performed using GraphPad Prism version 6 for Windows.

### Histopathology

Post-mortem tissues, including liver, spleen, kidneys, lungs, heart, brain, and the infected footpad, were collected 20 days post-infection, fixed in 10% formaldehyde, and embedded in paraffin. The footpads were descaled in 7% nitric acid (48 h), fixed with 10% formaldehyde, and embedded in paraffin. The organs and footpads of animals in the PBS group were collected as negative controls. Embedded tissue sections (3-μm-thick) were stained with hematoxylin and eosin (H&E) and periodic acid–Schiff (PAS) for observation by light microscopy using an Olympus BX51 microscope (Tokyo, Japan) equipped with an Olympus SC100 camera.

### *Sporothrix* antigens

Exoantigens from the mycelial phase of 14 isolates of the *Sporothrix* species (Table 1) were obtained in Sabouraud broth at 25°C using a rotary shaker (Multitron II—Infors HT, Switzerland) with constant orbital agitation (100 rpm) for 10 days as described previously [31]. Whole cell extracts of *Sporothrix* yeast cells (*S*. *brasiliensis* CBS 132990 and *S*. *schenckii* CBS 132974) were obtained as described elsewhere [32]. Protein extracts from isolates CBS 132990 (Ss54) and CBS 132974 (Ss118) were selected as references for immunoblot assays because they had been successfully used to diagnose sporotrichosis in ELISA and immunoblot [33, 34]. The protein concentrations were determined for all antigenic preparations using the Bradford method [35]. Exoantigens and whole cellular extracts were kept at -80°C until use.

### SDS-PAGE and immunoblotting assay

In order to evaluate the diversity of proteins secreted by different *Sporothrix* isolates, 14 exoantigens (5 μg/lane) were separated by SDS-PAGE using 10% gels [36] and silver-stained [37]. The relative molecular weights of the fractions were estimated using standard broad-range molecular weight markers (Protein Benchmark, Invitrogen). Individual bands were recorded, converted into binary data, and the profile of each *Sporothrix* exoantigen compared using Jaccard's similarity coefficient. Dendrogram analyses were performed in SYSTAT 13 (Systat Software, San Jose, CA).

Immunoblotting was carried out by resolving the 14 exoantigens and whole cellular proteins from two extracts (Ss54 = CBS 132990 and Ss118 = CBS 132974) on 10% SDS-PAGE, followed by electrotransfer to nitrocellulose membrane (0.2 μm; Bio-Rad) at 20 V for 30 min in transfer buffer (25 mM Tris base, 192 mM glycine, 20% methanol, pH 8.3) [38] using a Trans-Blot SD semi-dry device (Bio-Rad). Free binding sites were blocked overnight with PBS blocking buffer (1% bovine serum albumin supplemented with 0.05% [vol/vol] Tween 20, 5% [wt/vol] skim milk, pH 7.6) at 4°C. The membrane was cut lengthwise into 0.5-cm strips, and each blot was probed with its respective antisera at a dilution of 1:200 (PBS-Tween 20, 0.005%) for 1 h at room temperature. The membranes were washed three times with Tris-buffered saline (pH 7.5) containing 0.05% [vol/vol] Tween-20 for 10 min. Immunoreactive proteins were detected by incubation with peroxidase-conjugated goat anti-mouse IgG (1:1000 dilution) as secondary antibody (Sigma-Aldrich, USA) for 1 h at room temperature. Next, the membranes were washed with Tris-buffered saline (pH 7.5) containing 0.05% [vol/vol] Tween-20 and the signal detected using an enhanced chemiluminescence detection kit (GE Healthcare). Blots were imaged in a transilluminator (Uvitec Cambridge). Allience 4.7 software was used to take several images at different exposures, from 2 s each to a total of 10 images over 2 s.

### Two-dimensional gel electrophoresis (2DGE) of *S*. *brasiliensis* antigens and 2DGE immunoblotting assay

Proteins (300 μg) of *S*. *brasiliensis* Ss54 (= CBS 132990) were precipitated using the 2D clean-up kit (GE Healthcare, Piscataway, NJ, USA) following the manufacturer's recommendations. Proteins were mixed with IPG rehydration buffer (7 M urea, 2 M thiourea, 2% CHAPS, 1.2% DeStreak, 2% vol/vol isoelectric focusing [IEF] buffer pH 4–7, and trace bromophenol blue) to a final volume of 250 μl. The strips (pH 4–7, 13 cm) were allowed to rehydrate at 30 V for 12 h, focused (Ettan IPGphor III system; GE Healthcare, USA), equilibrated, apposed to the second dimension (10%) gels, and run as described [39]. Proteins were developed with silver [37] or directly transferred to nitrocellulose membrane (0.2 μm; Bio-Rad) in the case of immunoblot analysis.

For immunoblotting, membranes were probed against mouse antisera at a dilution of 1:200 (PBS-Tween 20, 0.005%) for 2 h at room temperature in order to evaluate seroreactive spots related to 3-carboxymuconate cyclase as described earlier by our group [34, 39]. Immunoblot conditions and immunodetection were essentially as described above (SDS-PAGE and immunoblotting assay).

## Results

### Development of a subcutaneous model of murine sporotrichosis

**Fig 1:**
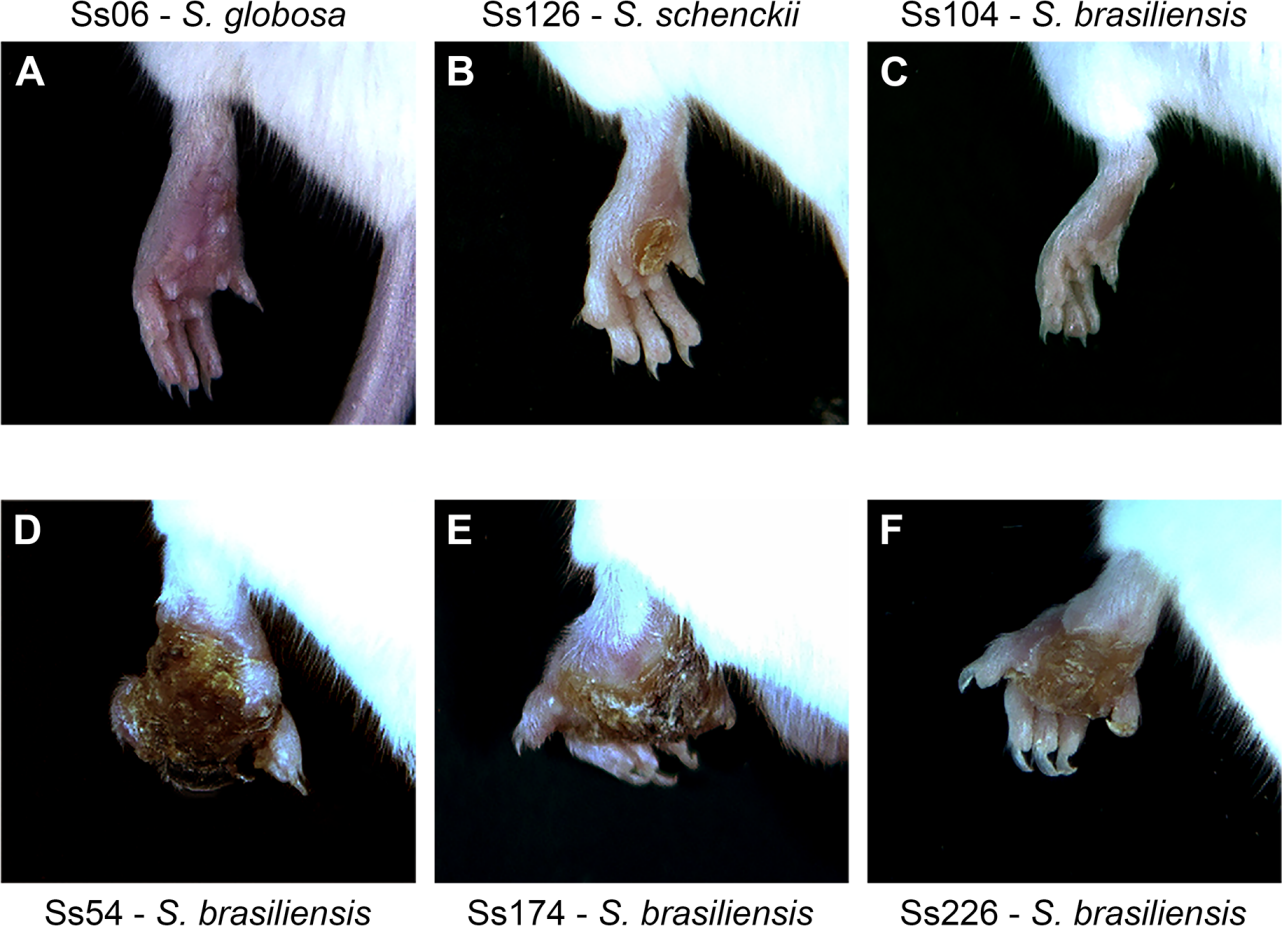
Murine model of subcutaneous infection by *Sporothrix* species shows isolate-specific lesion patterns. BALB/c male mice were inoculated in the left hind footpad with a high load of *Sporothrix* yeast cells (5×10^6^ cells/animal). (A–F) Left hind footpads of mice infected with *S*. *globosa* (Ss06), *S*. *schenckii* (Ss126), *S*. *brasiliensis* (Ss104), *S*. *brasiliensis* (Ss54), *S*. *brasiliensis* (Ss174), or *S*. *brasiliensis* (Ss226). Mice were imaged 27 days post-infection using the L-Pix Touch (Loccus Biotecnologia, São Paulo, Brazil) imaging system.

After challenge, lesion patterns in mice mimicked those of cats and humans, with intensities varying in an isolate-dependent manner from minor injuries to suppurating subcutaneous nodules that progressed proximally along lymphatic channels (Fig 1). Isolates Ss39 (*S*. *schenckii*), Ss126 (*S*. *schenckii*), Ss34 (*S*. *brasiliensis*), and Ss67 (*S*. *brasiliensis*) were able to develop minor injuries in the left hind footpad of infected mice. *S*. *brasiliensis* isolates Ss54, Ss66, Ss99, Ss174, Ss226, Ss252, Ss261, and Ss265 induced the development of large lesions, limiting the movement of the animals due to swelling and increased footpad size. In addition, *S*. *brasiliensis* isolates Ss252, Ss54, Ss99, Ss174, and Ss226 caused a partial loss of the fingers to complete footpad loss in some inoculated animals. Lesions were verified in the tails, back, and skin of 14.2% of infected animals. At the end of the study, animals that developed large lesions exhibited sequelae, usually in the footpad and the joint next to the region of the footpad (inoculum site). However, some isolates, such as the non-virulent Ss06 (*S*. *globosa*) and Ss104 (*S*. *brasiliensis*), did not result in lesions in the footpads. Notwithstanding, these non-virulent strains (in a subcutaneous route) were able to disseminate when subjected to an intravenous route during passages in BALB/c mice to restore the virulence, suggesting that virulence is affected by the chosen injection route.

### Virulence of *S*. *brasiliensis* is strain-dependent

**Fig 2:**
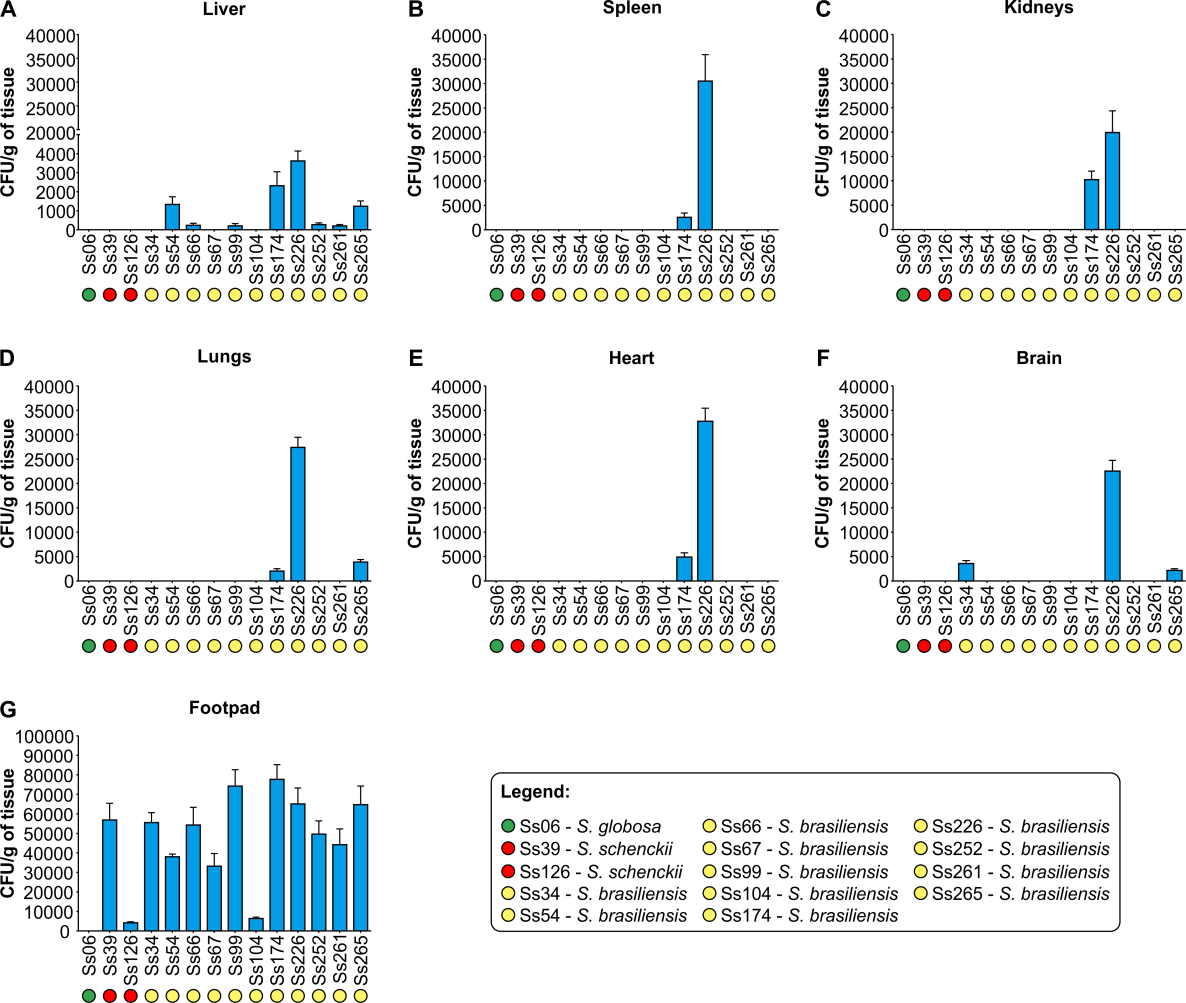
CFUs per organ in BALB/c male mice 20 days post-infection. All isolates were recovered from the initial site of inoculation into the left hind footpad (G), except for the non-virulent Ss06 (*S*. *globosa*). Greater dissemination, especially to the liver, was observed for strains belonging to the *S*. *brasiliensis* clade compared to *S*. *schenckii* and *S*. *globosa*. (A–G) Data are expressed as CFU/g of tissue in the liver, spleen, kidneys, lungs, heart, brain, and left hind footpad. All groups were significantly different by pairwise comparison. Statistical analysis is presented in the S1 Table.

The fungal tissue burden in the liver, spleen, kidneys, lungs, heart, brain, and footpad is shown in Fig 2. All *Sporothrix* isolates were recovered from the left hind footpad, except for *S*. *globosa* (Ss06; *P* < 0.001; Fig 2G). The dissemination power among *Sporothrix* isolates followed a subcutaneous route. After the footpad, the liver was the most affected organ (n = 8), followed by the lungs (n = 3), brain (n = 3), spleen (n = 2), heart (n = 2), and kidneys (n = 2). Across the main isolates in the pathogenic clade, strains belonging to the *S*. *brasiliensis* clade were usually more virulent than those belonging to *S*. *schenckii* and *S*. *globosa*. Among *S*. *brasiliensis* strains, Ss174 (*MAT1-1*) and Ss226 (*MAT1-2*) were significantly more virulent (*P* < 0.01) with high fungal burden and dissemination power, closely followed by Ss34 (*MAT1-1*), Ss54 (*MAT1-1*), Ss66 (*MAT1-2*), Ss99 (*MAT1-2*), Ss252 (*MAT1-2*), Ss261 (*MAT1-1*), and Ss265 (*MAT1-1*), whereas Ss67 (*MAT1-2*) and Ss104 (*MAT1-2*) were the least virulent strains, causing self-limited infection (footpad only) with lower invasiveness, similar to isolates Ss39 (*MAT1-2*) and Ss126 (*MAT1-2*) of *S*. *schenckii* (Fig 2). This suggests that the invasiveness of *S*. *brasiliensis* is strain-dependent, and mating type idiomorphs being directly associated with virulence in the *Sporothrix* spp. is unlikely. The isolate Ss06 (*S*. *globosa*, *MAT1-1*) was considered non-pathogenic because it was not able to colonize any organ. No fungal load was observed in organs from animals that received sterile PBS (Fig 2).

**Fig 3:**
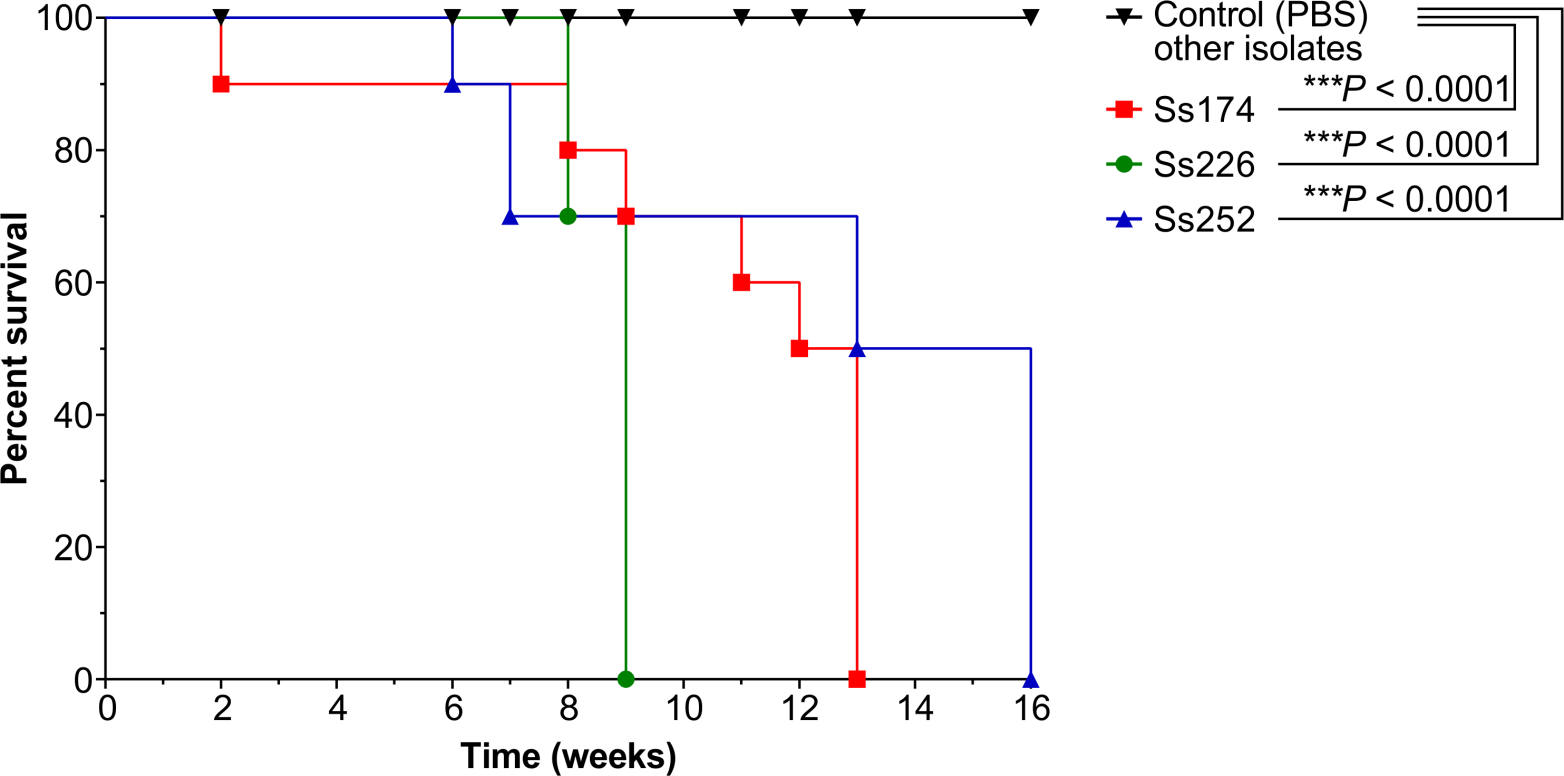
Survival curves for BALB/c mice inoculated subcutaneously with *Sporothrix* species or PBS (control group). All mice inoculated with highly invasive *S*. *brasiliensis* isolates (Ss174, Ss226, and Ss252) died between week 2 and 16 post-infection. ***P < 0.001 for survival in *S*. *brasiliensis* Ss174 (red line), Ss226 (green line), and Ss252 (blue line) compared to the control group (black line). Remaining *S*. *brasiliensis* (Ss34, Ss54, Ss66, Ss67, Ss99, Ss104, Ss261, and Ss265), *S*. *schenckii* (Ss39 and Ss126) and *S*. *globosa* (Ss06) infected groups (black line) behaved similarly to the PBS group and no animal died until the end of the assay.

The survival time of each mouse was recorded (Fig 3), and differences in the median survival time among strains were analyzed. All mice challenged with highly invasive strains (Ss226 [weeks 2–9], Ss174 [weeks 11–13], and Ss252 [weeks 6–16]) died between 2 and 16 weeks post-infection (Fig 3), although the numbers of CFUs were significantly greater in mice infected with Ss226 (*P* < 0.001) (Fig 2). On the other hand, mice challenged with strains Ss06, Ss34, Ss39, Ss54, Ss66, Ss67, Ss99, Ss104, Ss126, Ss261, and Ss265 were still alive after 16 weeks.

### Highly virulent *S*. *brasiliensis* induces critical weight loss in infected animals

**Fig 4:**
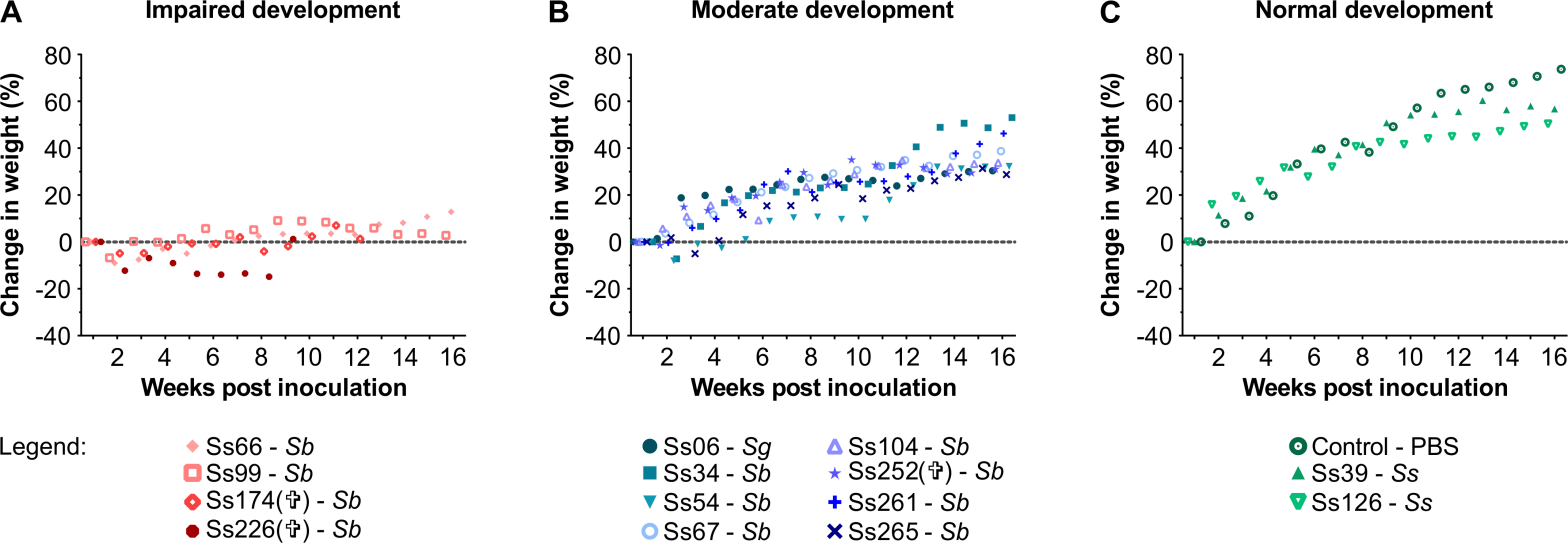
Highly virulent *S*. *brasiliensis* isolates induce critical weight loss leading to impaired development and death of infected animals. Data are expressed as percent weight loss (%) determined by measuring the animal’s weight every week post-inoculation and comparing them to the weight of the animal on the day of inoculation. (A) Significant weight loss in *S*. *brasiliensis* Ss66, Ss99, Ss174, and Ss226-infected groups, leading to the death of animals (Ss174 and Ss226) and impaired development compared to the PBS group (*P* < 0.0001). (B) Moderate development for *S*. *brasiliensis* Ss34, Ss54, Ss67, Ss104, Ss252, Ss261, and Ss265 and the *S*. *globosa* Ss06-infected group. Despite initial weight loss in early infection, mice began to regain weight between the 2^nd^ and 5^th^ week post-inoculation, but this evolution was not similar to the PBS group (*P* < 0.0001). (C) Normal development for *S*. *schenckii* Ss39 and Ss126-infected groups, similar to the PBS group, demonstrating the appearance and spontaneous resolution of *Sporothrix* infection. Statistical analysis is presented in the S2 Table.

The animals were evaluated for percentage weight loss during the survival experiments. We used weight loss as a virulence measure because it is correlated with mortality rate. Remarkably, mice inoculated with highly virulent isolates Ss174 (*P* < 0.0001) and Ss226 (*P* = 0.0006) induced substantial and chronic weight loss (-5% and -15% of their body weight, respectively) of infected animals (Fig 4A). In addition, mice infected with S. *brasiliensis* Ss34, Ss54, Ss67, Ss104, Ss252, Ss261, and Ss265 presented with moderate weight loss (*P* < 0.0001) compared to the non-infected group, and the mice began to regain weight between 2 and 5 weeks post-inoculation (Fig 4B). In contrast, *S*. *schenckii* isolates Ss126 (*P* = 0.0063) and Ss39 (*P* = 0.0519) did not induce weight loss in infected mice and the later did not reach the threshold (*P* < 0.05) for significance compared to the PBS group (Fig 4C).

### Histopathology

**Fig 5:**
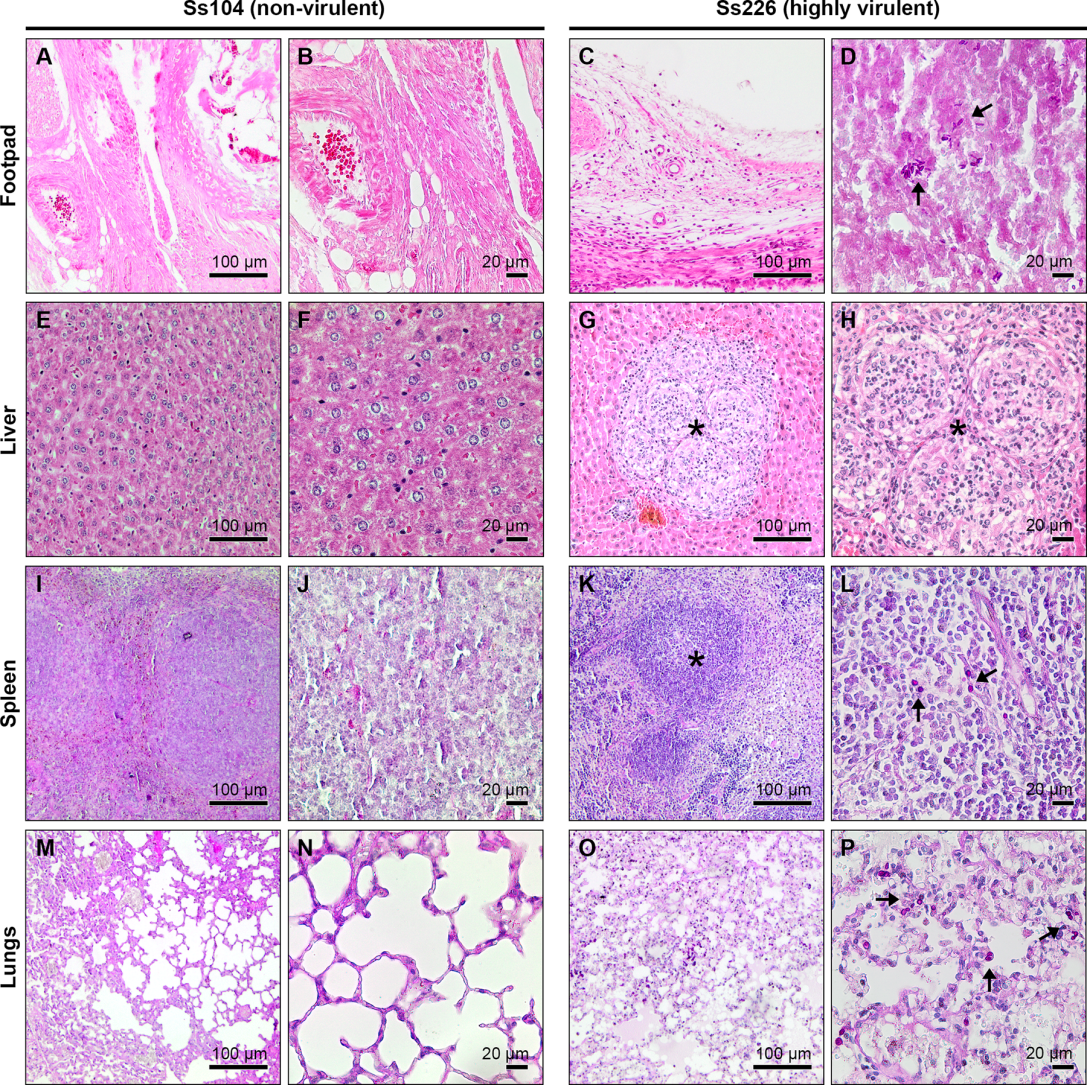
Histological patterns of isolates of *Sporothrix brasiliensis* exhibiting opposite virulence profiles. Periodic acid-Schiff-stained sections from the footpad (A–D), liver (E–H), spleen (I–L), and lungs (M–P) of mice infected with non-virulent (Ss104, left) and highly virulent (Ss226, right) *S*. *brasiliensis* isolates. Virulent isolates induced granuloma in the liver with mononuclear and polymorphonuclear leukocyte infiltrates (G and H, asterisk). Non-virulent isolate Ss104 presented normal histological characteristics (E and F) similar to the PBS group (control group). *Sporothrix* yeast cells can be seen in the footpad (D), spleen (L), and lungs (P) of mice infected with isolate Ss226 (arrow).

At the site of inoculation, histopathological parameters necrosis, suppurative inflammation, and mononuclear inflammatory infiltrate were noted in all *Sporothrix*-inoculated animals. All histopathological parameters increased in severity in *S*. *brasiliensis* isolates Ss174 and Ss226. However, a few strains were able to disseminate and induce granuloma (Ss99, Ss174, Ss226, and Ss261) and mononuclear/polymorphonuclear inflammatory infiltrates (Ss226, Ss252, ad Ss265) in the liver of infected animals (Fig 5). In the heart, an inflammatory infiltrate was observed in mice inoculated with Ss252. Fig 5 shows some of the differences in the histopathological parameters in the course of infection with two polar *S*. *brasiliensis* strains. Table 2 summarizes the virulence characteristics, weight loss, and histopathological features of *Sporothrix* isolates.

**Table 2:** Virulence characteristics of *Sporothrix* isolates based on the murine model of subcutaneous sporotrichosis.

Isolate	Species	Dissemination power^1^	Induction to death^2^	Development^3^	Virulence level
Ss06	*S*. *globosa*	None	No	Moderate development	Non-virulent
Ss39	*S*. *schenckii*	Footpad	No	Normal development	Low virulence
Ss126	*S*. *schenckii*	Footpad	No	Normal development	Low virulence
Ss67	*S*. *brasiliensis*	Footpad	No	Moderate development	Low virulence
Ss104	*S*. *brasiliensis*	Footpad	No	Moderate development	Low virulence
Ss34	*S*. *brasiliensis*	Footpad and brain	No	Moderate development	Medium virulence
Ss54	*S*. *brasiliensis*	Footpad and liver	No	Moderate development	Medium virulence
Ss66	*S*. *brasiliensis*	Footpad and liver	No	Impaired development	Medium virulence
Ss99	*S*. *brasiliensis*	Footpad and liver	No	Impaired development	Medium virulence
Ss261	*S*. *brasiliensis*	Footpad and liver	No	Moderate development	Medium virulence
Ss252	*S*. *brasiliensis*	Footpad and liver	Yes	Moderate development	High virulence
Ss265	*S*. *brasiliensis*	Footpad, liver, lungs, and brain	No	Moderate development	High virulence
Ss174	*S*. *brasiliensis*	Footpad, liver, spleen, kidneys, lungs, and heart	Yes	Impaired development	High virulence
Ss226	*S*. *brasiliensis*	Footpad, liver, spleen, kidneys, lungs, heart, and brain	Yes	Impaired development	High virulence

^1^Based on colony-forming unity assay

^2^Based on survival rates

^3^Based on weight loss rates.

### Connecting virulence, protein secretion, and immunogenicity

**Fig 6:**
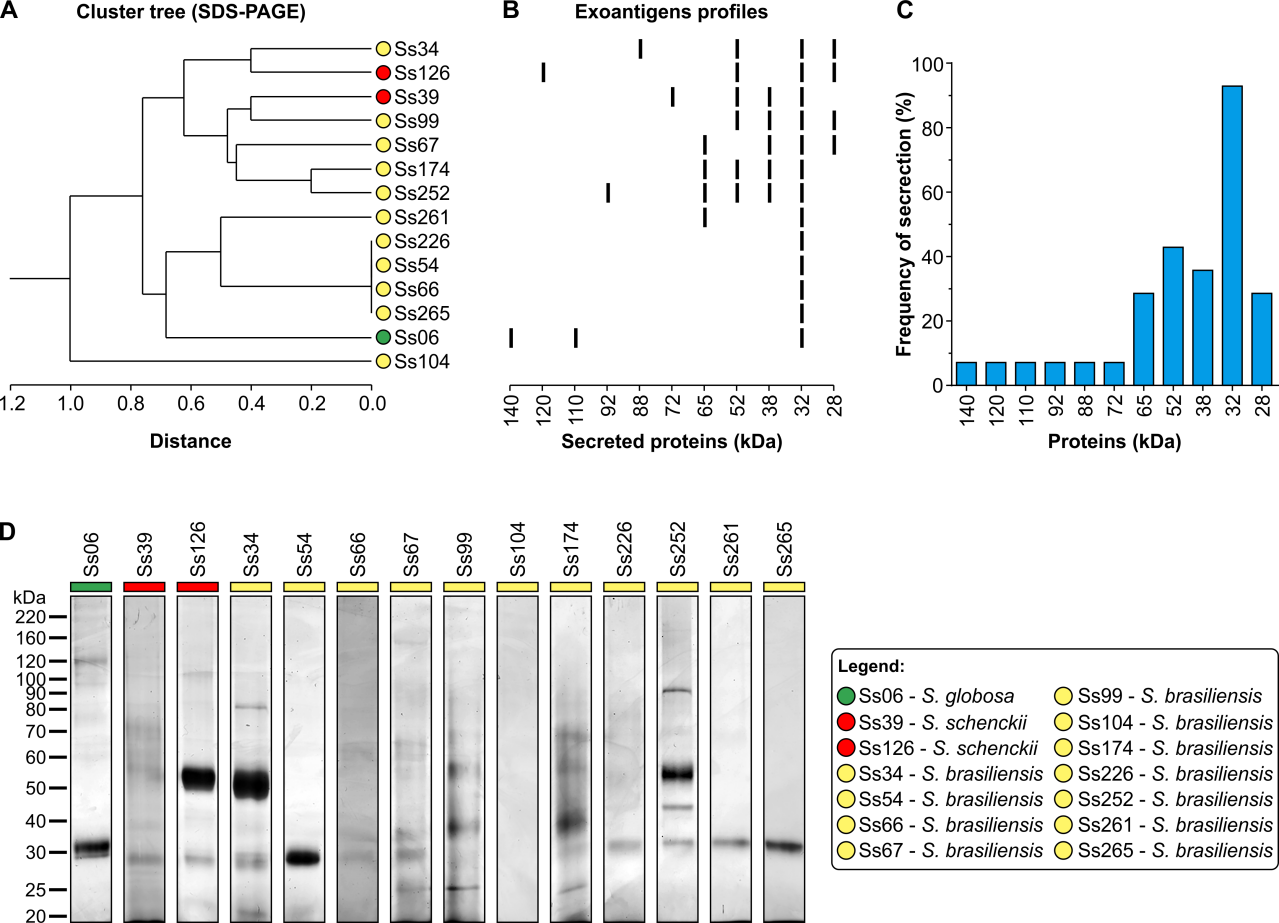
Diversity of secreted proteins in the *Sporothrix* spp. (A) Dendrogram showing the exoantigen banding patterns of 14 *Sporothrix* strains. Clustering was performed according to Jaccard's similarity coefficient based on protein bands developed from 140 to 28 kDa. *Sporothrix brasiliensis* (yellow), *S*. *schenckii* (red), and *S*. *globosa* (green) species are indicated close to the isolate code. (B) The banding patterns of secreted proteins according to 1D SDS-PAGE. Molecular weights are shown at the bottom. (C) Frequency and diversity of secreted proteins showing a major band of 32 kDa secreted by all isolates except *S*. *brasiliensis* Ss104. (D) Secretome profile of *Sporothrix* spp. after 10 days of incubation at 25°C and 100 rpm in Sabouraud broth, showing the abundance and diversity of proteins. The exoantigens (5 μg/lane) were resolved by 1D SDS-PAGE and the proteins developed by silver staining. The molecular masses of standard proteins are given to the left of the gel in kDa (Bench Mark Protein Ladder, Invitrogen). Exoantigens were obtained in three biological replicates.

The protein secretion profiles of the *Sporothrix* strains analyzed by SDS-PAGE were heterogeneous, but important differences were observed regarding localization and the intensity of several bands (Fig 6A and 6B). More than 11 bands were stained (Fig 6D), and the proteins present in all strains corresponded to molecular weights of 140, 120, 110, 92, 88, 72, 65, 52, 38, 32, and 28 kDa. Each strain exhibited a major band at 32 kDa (Fig 6C).

**Fig 7:**
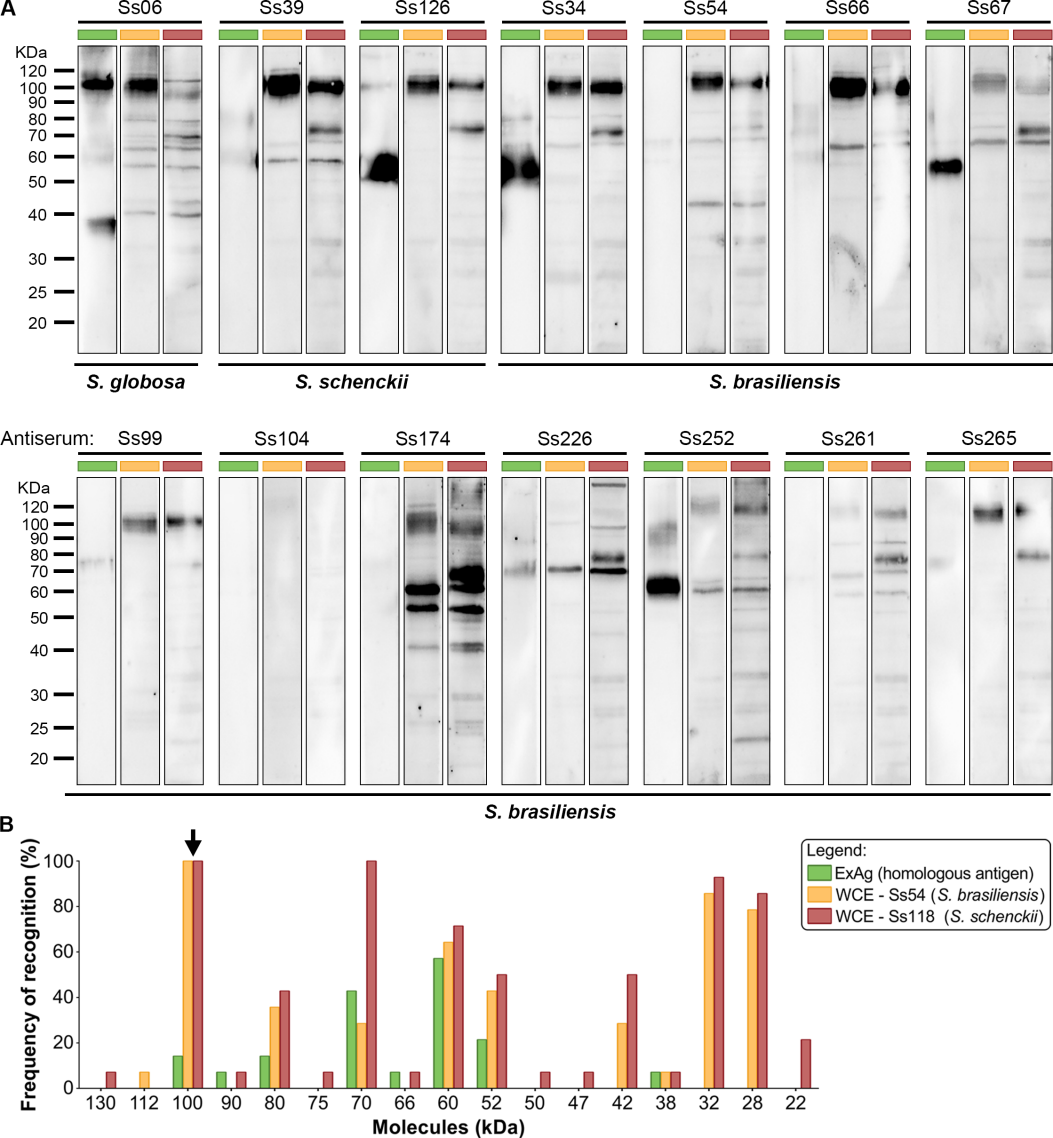
Humoral diversity in experimental sporotrichosis. (A) Immunoblot patterns of clinical isolates of *Sporothrix* using exoantigen (green bar) and reference whole cell extracts derived from *S*. *brasiliensis* (Ss54 = CBS 132990; yellow bar) and *S*. *schenckii* (Ss118 = CBS 132974; red bar). Fungal proteins were resolved on 10% SDS-PAGE, electrotransferred to PVDF membranes, and probed against a pooled mouse serum (homologous antisera n = 5, diluted at 1:200). (B) Frequency and diversity of immunoreactive proteins in experimental sporotrichosis revealed immunodominant molecules in the 100-kDa fractions and oscillating between 60 and 70-kDa (gp60-70).

To further examine the contribution of immunogenicity, an immunoblotting reaction employing the *Sporothrix* polyclonal antiserum revealed that IgG antibodies produced by the mice recognized 17 fractions overall, ranging from 22 to 130kDa in the whole cell extracts and from 38 to 100 kDa in the exoantigen preparations. Notably, immunodominant molecules included 3-carboxymuconate cyclase (KP233225, a classical glycoprotein oscillating between the 60 and 70 kDa fractions) and against a 100-kDa molecule (KP247558) in nearly 100% of the cases (Fig 7). Moreover, the antigenic profiles were isolate-specific, supporting the hypothesis that different *Sporothrix* can elicit a heterogeneous humoral response over time, but a high level of cross reaction was observed between *S*. *brasiliensis* and *S*. *schenckii* whole cell extracts and the same antiserum (Fig 7A), supporting that antigenic epitopes are conserved among different species embedded in the pathogenic clade.

**Fig 8:**
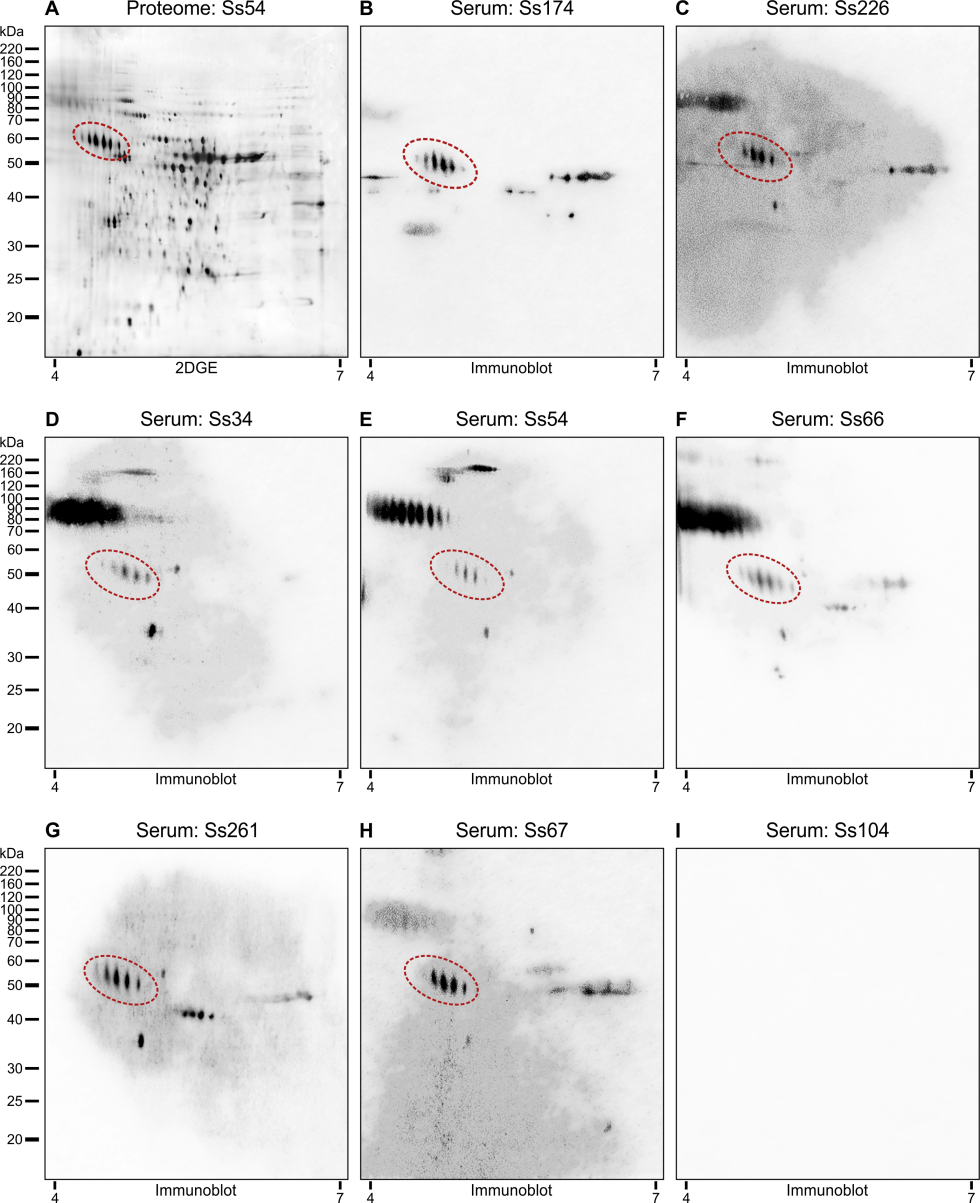
Immunoreactivity of *S*. *brasiliensis* Ss54 (= CBS 132990) proteins resolved by 2DGE with mice sera reveals high cross-reactivity and isoforms of gp60 as major component recognized. *S*. *brasiliensis* whole cellular extract (yeast phase) were separated by IEF at pH 4 to 7 in the first dimension, followed by 10% SDS-PAGE in the second dimension. Gels were subsequently silver stained (A) or immunoblotted (B-I) with a 1:200 dilution of infected mice sera indicated at the top. Red dot areas indicate all seroreactive protein spots identified as 3-carboxymuconate cyclase. Immunoblot assay using Ss104 antisera did not detect immunoreactivity (I), similar to the 1D assay. Acidic and basic ends are denoted at the bottom, and the positions of molecular mass markers (in kilodaltons) are indicated to the left of the gels series.

In a scenario were gp60-70 were the main antigen recognized via 1D immunoblot we explored 2D-immunoblot to confirm the identity of this antigen. In doing so, we used the same protein extract describe by Rodrigues *et al*. [39], which were resolved by 2DGE followed by immunoblot. Thereafter membranes were probed against a pool of sera of mice experimentally infected in this study. Multiple distinct seroreactive spots in the same gel were previously identified as the same protein (Fig 8A) and represent charge (isoforms) and molecular mass variants (glycoforms) [34, 39]; examples of these variants are circumscribed within a red area in Fig 8B–8I), confirming that isoforms of 3-carboxymuconate cyclase are recognized by IgG antibodies during experimental infection of *S*. *brasiliensis*. Small variations were noted in immunoblot profiling considering the recognition pattern using the crude proteome in 1D (crude Ss54; Fig 7A) and the precipitated proteome used for 2DGE (Ss54; Fig 8B–8I). Indeed, the 2DGE analysis of fungal proteins is quite difficult due to the high concentration of contaminant molecules. To remove interfering compounds from our crude extract (salts, lipids, detergents, nucleic acids and phenolic compounds) and improve 2DGE we used 2-D Clean-Up Kit as previously recommended [32, 39].

## Discussion

A pivotal challenge in studying the pathogenesis of *Sporothrix* species is to develop a quantifiable approach that reproduces both the pathophysiology and symptomology of sporotrichosis. Mice are often used to assess the virulence of *Sporothrix* species (BALB/c, C57BL/6, OF-1), but simple models varying from cultured mammalian cells [40] to the caterpillar *Galleria mellonella* [41, 42] have been employed successfully. We selected the murine system because endothermy seems to protect mammals from most *Sporothrix* spp. [26, 43]. We chose a set of human and animal-derived isolates of *S*. *brasiliensis* that are of both medical and veterinary interest and have been the main focus of extensive research in the last few years due to the global emergence of sporotrichosis [20, 43]. Mice challenged subcutaneously with *S*. *brasiliensis* developed typical lesions of sporotrichosis in the footpad 1 week post-infection; they very similar to those observed in human and animal sporotrichosis, with an ulcerous-crust appearance, validating our murine model. Moreover, histopathology showed granulomatous inflammation and a pyogenic process with the presence of yeasts similar to those observed by Brito *et al*. [14].

Previous models of murine sporotrichosis employed a subcutaneous route of infection and compared the virulence levels among several clinical isolates of *Sporothrix*, confirming differences among them [14, 16], especially interspecific variation as observed between *S*. *brasiliensis* and *S*. *schenckii* [15]. Previous epidemiological studies have shown that recombination is most likely to occur in *S*. *schenckii* which allows generation of greater genetic diversity [27]. On the other hand, this phenomenon is expected to be absent or less frequent in natural populations of *S*. *brasiliensis* resulting in highly clonal population structure during outbreaks [27]. Remarkably, our results support the heterogeneity of virulence levels in *S*. *brasiliensis* isolates with low, medium, and high virulence, as well as confirm the absence of virulence in *S*. *globosa* as reported in previous studies of a systemic route of infection [8, 9]. Another striking finding is that *S*. *brasiliensis* (Ss34, Ss174, and Ss226) can disseminate across the central nervous system, even when we employed a subcutaneous route. These results involving brain dissemination parallel available data from a systemic model based on CFUs [9], histological analysis, or a molecular approach using species-specific PCR [24]. Such atypical manifestation in the central nervous system may draw the attention of physicians to the occurrence of more severe manifestations of disease caused by *Sporothrix* in endemic areas [44, 45]. Moreover, animal-born isolates (Ss54, Ss174, Ss226, and Ss252) presented greater virulence than the remaining *S*. *brasiliensis* isolates of human origin. This emerging virulence could explain the recent appearance of atypical and more severe cases of sporotrichosis during zoonotic episodes in areas where *S*. *brasiliensis* is endemic [46, 47].

Based on a systemic route of infection, Arrillaga-Moncrieff *et al*. [9] and Fernandes *et al*. [8] compared the virulence of different species of the *Sporothrix* and found that *S*. *brasiliensis* was the most virulent, followed by *S*. *schenckii* and *S*. *globosa*, which had low or no pathogenicity. In our study, it was clear that the dissemination power is related to the route of infection, as the same isolates previously found to be highly virulent (Ss54), medium (Ss126), and non-virulent (Ss06) in a systemic model of infection [8] presented with a lower capacity for dissemination when submitted to a subcutaneous route (Fig 2). We emphasize that the subcutaneous route of infection reflects the natural route of transmission. Moreover, when a cat transmits the fungus by scratching or biting, the contamination is caused by the yeast inoculum and not by conidia [20], a morphotype recognized to be more virulent to mammals [48]. This may enhance the alternative, feline-driven transmission route [3]. Therefore, we used 5 × 10^6^ yeast cells as inoculum considering the high yeast load present in the skin lesions and exudates of cats [19].

Within the intraspecific diversity *S*. *brasiliensis*, we identified isolates Ss226 and Ss174 as highly virulent. The ability of these strains to cause earlier death than remaining *Sporothrix* could be related to their capacity to induce liver damage, thereby impairing or leading to earlier loss of liver function. Histological parameters revealed granuloma formation and mononuclear/polymorphonuclear infiltration in isolates that induced dramatic weight loss and death. These data reinforce the high invasiveness of some strains of *S*. *brasiliensis* compared to our controls, *S*. *schenckii* and *S*. *globosa*. On the other hand, we recognize Ss104 as a low virulent *S*. *brasiliensis* strain. Remarkably, this isolate was recovered from a human case nearly 2,000 km from the epicenter of the cat-transmitted epidemic in the metropolitan area of Rio de Janeiro, Brazil [27]. The minimum genome size of isolate Ss104 was estimated to be 31.3 Mb by pulsed field gel electrophoresis [23], comprising at least six chromosomal bands (7.0, 6.7, 5.8, 5.4, 3.5, and 2.9 Mb), which deviates genetically from the highly virulent clone of *S*. *brasiliensis* implicated in the Rio de Janeiro epidemic that usually presents with five chromosomal bands and has an average genome size of 25.7 Mb. Moreover, gene synteny highlighted differences in isolate Ss104, including gene translocations and a considerable amount of repetitive DNA sequences [23], which may be related to differing genomic organization and explain the atypical phenotype of virulence. Among human pathogenic fungi, such as *Blastomyces* spp. [49], *Histoplasma capsulatum* [50], and *Paracoccidioides* spp. [51], isolates with a divergent genetic repertoire have been demonstrated to present with differential regulation of critical virulence factors, affecting pathogenicity to the mammalian host.

*Sporothrix* is a heterothallic fungus with a single mating type locus that produces two alleles, *MAT1-1* and *MAT1-2*. This system requires two compatible partners for mating to occur [29], though the sexual cycle in clinical *Sporothrix* has not yet been observed in nature or under laboratory conditions. Interestingly, *S*. *brasiliensis* populations associated with feline epizooties have been suggested to reproduce clonally with the predominance of *MAT1-1* or *MAT1-2* in South and Southeast Brazil, respectively [29]. Therefore, we explored the virulence profiles among distinct mating type idiomorphs to test whether there is a connection between mating type and virulence in *Sporothrix*. Interestingly, the most virulent isolates harbor opposite mating types (i.e., Ss174 = *MAT1-1*; Ss226 = *MAT1-2*), suggesting that both are similarly pathogenic with high dissemination power, induction of weight loss, and induction of death. This is consistent with the severity of the disease observed among animals naturally infected with *S*. *brasiliensis* in both epidemics occurring in South and Southeast Brazil [20–22]. Mating type has been found to be a source of variation in virulence in certain serotypes of *Cryptococcus neoformans* [52] and *Aspergillus fumigatus* [53], including a deviating ratio between **a**/α or *MAT1-1*/*MAT1-2* recovered from clinical cases. On the other hand, mating type appears to have no influence in *Histoplasma capsulatum*, in which both mating types are evenly distributed in the soil (1:1), deviating in clinical samples with the majority of *MAT1-1* (7:1) [54, 55], but no significant difference was found between strains *MAT1-1* and *MAT1-2* in a murine model of infection [56], similar to our data in clinical *Sporothrix*.

*Sporothrix*-mammal interactions may result in different outcomes varying from self-limited to severe disseminated infection. Such variation may be regarded as a function of the host's immune response delivered against *Sporothrix*. Remarkably, the consequence of this complex interaction may reduce the amount of continuing damage caused by the microbe to a level that is insignificant [57]. The cell-mediated immunity during sporotrichosis involves interactions between CD4^+^ T cells, macrophages, dendritic cells, neutrophils and the release of soluble mediators which are protective and promote clearance of the infection. Of these cell populations, CD4^+^ T cells and macrophages play an essential role for resolution of sporotrichosis, since athymic nude mice clearly demonstrate that loss of CD4^+^ T cells, but not CD8+ T cells, renders mammals susceptible to *Sporothrix* infection [16]. In this scenario, it is tempting to hypothesize that the different lesion patterns and disease severity observed in our study may reflect distinct lymphocyte activation profiles by the different strains used. To further reinforce this notion, it was recently demonstrated that *S*. *brasiliensis* and *S*. *schenckii* are differentially recognized by human peripheral blood mononuclear cells, depending on the morphotype (conidia, germlings or yeasts) and cell wall composition [58].

To assess the humoral response against *Sporothrix* species, mice have been widely used in experimental sporotrichosis, as well as serum derived from naturally infected humans [59, 60] and cats [33, 34]. Cellular and humoral immune responses triggered upon *Sporothrix* introduction into the subcutaneous tissue may play important roles in the development and severity of sporotrichosis [61–63]. We reported previously that *Sporothrix* molecules are able to induce an important humoral response during human [39], murine [8], and feline sporotrichosis [34]. Here, the notion that divergent strains elicit variant-specific humoral immunity is supported by the finding that most *S*. *brasiliensis* strains induced antibodies against a great diversity of antigens during murine sporotrichosis, a scenario similar to the ongoing epizootics in Rio de Janeiro. *S*. *brasiliensis*-infected cats also produce a diversity of antibodies that seem to be divergent from one diseased cat to other [34], but this diverse repertoire of antibodies generates high levels of cross reactivity when probed against *S*. *brasiliensis* and *S*. *schenckii* antigenic preparations, supporting a conservation of epitopes across the main pathogenic species [34, 39]. Judging from these immunogenic profiles, our results may also shed light on recent evolutionary divergence in the clinical clade, as this recognition pattern is absent in the non-virulent ancestral *S*. *mexicana* [39], a species inserted in the *S*. *pallida* complex with attenuated virulence to mammals [9, 11].

Despite the diversity in the clinical clade, we found that 3-carboxymuconate cyclase (Accession number KP233225; gp60 in *S*. *brasiliensis* and gp70 in *S*. *schenckii*) is the major antigenic target in the early IgG response of murine, feline [34], and human sporotrichosis [39] whereby, after infection, more and more gp60-70-specific antibodies can be found [63]. Classically, gp60-70-specific antibodies are associated with protective immunity in which passive immunization with either polyclonal monospecific or monoclonal antibodies raised against 3-carboxymuconate cyclase can reduce the fungal burden of infected animals, probably by inhibiting adhesion to the components of the extracellular matrix and enhancing the opsonization of yeasts [17, 64–68].

Judging from a highly polymorphic humoral response raised against strain-specific immunity, we highlight gp60-70 as a common molecule recognized by all antisera of *Sporothrix*-infected mice. Therefore, we propose gp60-70 as an interesting candidate to match all pathogenic *Sporothrix*, including those associated with the most severe disease, as in *S*. *brasiliensis*, probably inducing cross-protection against a wide range of antigenic variants. The relevance of 3-carboxymuconate cyclase as a target in the humoral response has been shown in several studies [15, 63, 64, 66, 67, 69, 70]. Other important *Sporothrix* vaccine targets include a 100-kDa antigenic molecule. A 100-kDa molecule has also been found in the human immunoproteome (Accession number KP247558) and identified as endoplasmic signal peptidase. Similar studies exploring the humoral response in sporotrichosis have demonstrated an antigen-specific IgG response against a peptide hydrolase (44 kDa) and an enolase (47 kDa). Specific antibodies were able to significantly enhance phagocytosis, inhibit adhesion, affording *in vivo* protection [71]. Taken together, these results are helping build an important panel of candidate vaccine antigens with the potential for immunization in areas of hyperendemicity. Thus, understanding the diversity of antigenic variation may help in designing vaccines that represent the worldwide repertoire of polymorphic *Sporothrix* antigens.

In conclusion, our data broaden the current view on *S*. *brasiliensis* epidemics driven by populations with predominantly low levels of genetic diversity [27]. We demonstrate that the virulence process is strain-specific. Mimicking natural infection via traumatic inoculation, we demonstrated that the route is crucial to understanding the pathogenesis of sporotrichosis. We also demonstrated the presence of a panel of immunodominant molecules that could be used as antigens in anti-*Sporothrix* vaccines. These advances have opened up important possibilities for comparative genomic studies, characterization of new *Sporothrix* antigens, and confirming vaccine targets to tackle the progress of sporotrichosis in endemic areas.

## Supporting information

S1 Table Statistical analysis of the colony forming units (CFU) assay. Results were compared among groups and analyzed by analysis of variance (ANOVA) followed by post-hoc Tukey. *P*≤0.05 was considered significant. All analyses were performed using GraphPad Prism version 6 for Windows.
(DOCX)

S2 Table Statistical analysis of the weight loss assay. The percent weight loss was determined by measuring each animal’s weight every week post-inoculation (up to 16 weeks) and comparing it to the animal’s weight on the day of inoculation. Data were analyzed by paired t-test. *P*≤0.05 was considered significant. All analyses were performed using GraphPad Prism version 6 for Windows.
(DOCX)
