# Supplementary material for: Exploring virulence and immunogenicity in the emerging pathogen Sporothrix brasiliensis
Source: PLoS Negl Trop Dis. 2017 Aug 30;11(8):e0005903. doi: 10.1371/journal.pntd.0005903 (PMC5595342; doi:10.1371/journal.pntd.0005903)
Supplement: S1 Table — Results were compared among groups and analyzed by analysis of variance (ANOVA) followed by post-hoc Tukey. P≤0.05 was considered significant. All analyses were performed using GraphPad Prism version 6 for Windows. (DOCX) [file pntd.0005903.s001.docx]

**S1 Table.** Statistical analysis of the colony forming units (CFU) assay. Results were compared among groups and analyzed by analysis of variance (ANOVA) followed by post-hoc Tukey. *P*≤0.05 was considered significant. All analyses were performed using GraphPad Prism version 6 for Windows.

| **Organ/member** | **Comparison** | **Significant?**  ***P* < 0.05?** | **Summary** | **95% CI of diff** |
| --- | --- | --- | --- | --- |
| Foot | Ss39 vs Ss126 | Yes | *** | 38960 to 66490 |
|  | Ss39 vs Ss34 | No | ns | -12390 to 15140 |
|  | Ss39 vs Ss54 | Yes | *** | 5103 to 32640 |
|  | Ss39 vs Ss66 | No | ns | -11170 to 16360 |
|  | Ss39 vs Ss67 | Yes | *** | 9970 to 37500 |
|  | Ss39 vs Ss99 | Yes | ** | -31100 to -3568 |
|  | Ss39 vs Ss104 | Yes | *** | 36740 to 64270 |
|  | Ss39 vs Ss174 | Yes | *** | -34560 to -7027 |
|  | Ss39 vs Ss226 | No | ns | -21980 to 5557 |
|  | Ss39 vs Ss252 | No | ns | -6484 to 21050 |
|  | Ss39 vs Ss261 | No | ns | -1080 to 26450 |
|  | Ss39 vs Ss265 | No | ns | -21610 to 5920 |
|  | Ss126 vs Ss34 | Yes | *** | -65110 to -37580 |
|  | Ss126 vs Ss54 | Yes | *** | -47620 to -20090 |
|  | Ss126 vs Ss66 | Yes | *** | -63900 to -36360 |
|  | Ss126 vs Ss67 | Yes | *** | -42750 to -15220 |
|  | Ss126 vs Ss99 | Yes | *** | -83820 to -56290 |
|  | Ss126 vs Ss104 | No | ns | -15980 to 11550 |
|  | Ss126 vs Ss174 | Yes | *** | -87280 to -59750 |
|  | Ss126 vs Ss226 | Yes | *** | -74700 to -47170 |
|  | Ss126 vs Ss252 | Yes | *** | -59210 to -31670 |
|  | Ss126 vs Ss261 | Yes | *** | -53800 to -26270 |
|  | Ss126 vs Ss265 | Yes | *** | -74340 to -46800 |
|  | Ss34 vs Ss54 | Yes | ** | 3729 to 31260 |
|  | Ss34 vs Ss66 | No | ns | -12550 to 14990 |
|  | Ss34 vs Ss67 | Yes | *** | 8596 to 36130 |
|  | Ss34 vs Ss99 | Yes | ** | -32470 to -4942 |
|  | Ss34 vs Ss104 | Yes | *** | 35370 to 62900 |
|  | Ss34 vs Ss174 | Yes | *** | -35930 to -8401 |
|  | Ss34 vs Ss226 | No | ns | -23350 to 4183 |
|  | Ss34 vs Ss252 | No | ns | -7858 to 19670 |
| **Organ/member** | **Comparison** | **Significant?**  ***P* < 0.05?** | **Summary** | **95% CI of diff** |
|  | Ss34 vs Ss261 | No | ns | -2454 to 25080 |
|  | Ss34 vs Ss265 | No | ns | -22990 to 4546 |
|  | Ss54 vs Ss66 | Yes | ** | -30040 to -2509 |
|  | Ss54 vs Ss67 | No | ns | -8899 to 18630 |
|  | Ss54 vs Ss99 | Yes | *** | -49970 to -22440 |
|  | Ss54 vs Ss104 | Yes | *** | 17870 to 45400 |
|  | Ss54 vs Ss174 | Yes | *** | -53430 to -25900 |
|  | Ss54 vs Ss226 | Yes | *** | -40850 to -13310 |
|  | Ss54 vs Ss252 | No | ns | -25350 to 2179 |
|  | Ss54 vs Ss261 | No | ns | -19950 to 7584 |
|  | Ss54 vs Ss265 | Yes | *** | -40480 to -12950 |
|  | Ss66 vs Ss67 | Yes | *** | 7376 to 34910 |
|  | Ss66 vs Ss99 | Yes | *** | -33690 to -6161 |
|  | Ss66 vs Ss104 | Yes | *** | 34150 to 61680 |
|  | Ss66 vs Ss174 | Yes | *** | -37150 to -9621 |
|  | Ss66 vs Ss226 | No | ns | -24570 to 2964 |
|  | Ss66 vs Ss252 | No | ns | -9078 to 18460 |
|  | Ss66 vs Ss261 | No | ns | -3673 to 23860 |
|  | Ss66 vs Ss265 | No | ns | -24210 to 3326 |
|  | Ss67 vs Ss99 | Yes | *** | -54840 to -27300 |
|  | Ss67 vs Ss104 | Yes | *** | 13000 to 40540 |
|  | Ss67 vs Ss174 | Yes | *** | -58300 to -30760 |
|  | Ss67 vs Ss226 | Yes | *** | -45710 to -18180 |
|  | Ss67 vs Ss252 | Yes | ** | -30220 to -2688 |
|  | Ss67 vs Ss261 | No | ns | -24820 to 2717 |
|  | Ss67 vs Ss265 | Yes | *** | -45350 to -17820 |
|  | Ss99 vs Ss104 | Yes | *** | 54080 to 81610 |
|  | Ss99 vs Ss174 | No | ns | -17230 to 10310 |
|  | Ss99 vs Ss226 | No | ns | -4641 to 22890 |
|  | Ss99 vs Ss252 | Yes | *** | 10850 to 38380 |
|  | Ss99 vs Ss261 | Yes | *** | 16250 to 43790 |
|  | Ss99 vs Ss265 | No | ns | -4279 to 23250 |
|  | Ss104 vs Ss174 | Yes | *** | -85070 to -57530 |
|  | Ss104 vs Ss226 | Yes | *** | -72480 to -44950 |
|  | Ss104 vs Ss252 | Yes | *** | -56990 to -29460 |
| **Organ/member** | **Comparison** | **Significant?**  ***P* < 0.05?** | **Summary** | **95% CI of diff** |
|  | Ss104 vs Ss261 | Yes | *** | -51590 to -24050 |
|  | Ss104 vs Ss265 | Yes | *** | -72120 to -44590 |
|  | Ss174 vs Ss226 | No | ns | -1182 to 26350 |
|  | Ss174 vs Ss252 | Yes | *** | 14310 to 41840 |
|  | Ss174 vs Ss261 | Yes | *** | 19710 to 47250 |
|  | Ss174 vs Ss265 | No | ns | -819.5 to 26710 |
|  | Ss226 vs Ss252 | Yes | * | 1725 to 29260 |
|  | Ss226 vs Ss261 | Yes | *** | 7129 to 34660 |
|  | Ss226 vs Ss265 | No | ns | -13400 to 14130 |
|  | Ss252 vs Ss261 | No | ns | -8362 to 19170 |
|  | Ss252 vs Ss265 | Yes | * | -28900 to -1362 |
|  | Ss261 vs Ss265 | Yes | *** | -34300 to -6767 |
| Brain | Ss34 vs Ss226 | Yes | *** | -20940 to -16970 |
|  | Ss34 vs Ss265 | No | ns | -563.7 to 3414 |
|  | Ss226 vs Ss265 | Yes | *** | 18390 to 22370 |
| Heart | Ss174 vs Ss226 | Yes | *** | -30410 to -25290 |
| Lungs | Ss174 vs Ss226 | Yes | *** | -27260 to -23480 |
|  | Ss174 vs Ss265 | No | ns | -3741 to 45.53 |
|  | Ss226 vs Ss265 | Yes | *** | 21630 to 25410 |
| kidneys | Ss174 vs Ss226 | Yes | *** | -14010 to -5300 |
| Spleen | Ss174 vs Ss226 | Yes | *** | -32940 to -22940 |
| Liver | Ss54 vs Ss66 | Yes | *** | 419.4 to 1780 |
|  | Ss54 vs Ss99 | Yes | *** | 449.3 to 1810 |
|  | Ss54 vs Ss174 | Yes | *** | -1662 to -301.1 |
|  | Ss54 vs Ss226 | Yes | *** | -2966 to -1605 |
|  | Ss54 vs Ss252 | Yes | *** | 384.9 to 1746 |
|  | Ss54 vs Ss261 | Yes | *** | 453.4 to 1814 |
|  | Ss54 vs Ss265 | No | ns | -583.6 to 777.3 |
|  | Ss66 vs Ss99 | No | ns | -650.6 to 710.3 |
|  | Ss66 vs Ss174 | Yes | *** | -2762 to -1401 |
|  | Ss66 vs Ss226 | Yes | *** | -4066 to -2705 |
|  | Ss66 vs Ss252 | No | ns | -715.0 to 645.9 |
|  | Ss66 vs Ss261 | No | ns | -646.4 to 714.5 |
|  | Ss66 vs Ss265 | Yes | *** | -1683 to -322.5 |
|  | Ss99 vs Ss174 | Yes | *** | -2792 to -1431 |
| **Organ/member** | **Comparison** | **Significant?**  ***P* < 0.05?** | **Summary** | **95% CI of diff** |
|  | Ss99 vs Ss226 | Yes | *** | -4096 to -2735 |
|  | Ss99 vs Ss252 | No | ns | -744.8 to 616.0 |
|  | Ss99 vs Ss261 | No | ns | -676.3 to 684.6 |
|  | Ss99 vs Ss265 | Yes | *** | -1713 to -352.4 |
|  | Ss174 vs Ss226 | Yes | *** | -1985 to -623.7 |
|  | Ss174 vs Ss252 | Yes | *** | 1366 to 2727 |
|  | Ss174 vs Ss261 | Yes | *** | 1435 to 2796 |
|  | Ss174 vs Ss265 | Yes | *** | 397.9 to 1759 |
|  | Ss226 vs Ss252 | Yes | *** | 2671 to 4031 |
|  | Ss226 vs Ss261 | Yes | *** | 2739 to 4100 |
|  | Ss226 vs Ss265 | Yes | *** | 1702 to 3063 |
|  | Ss252 vs Ss261 | No | ns | -611.9 to 749.0 |
|  | Ss252 vs Ss265 | Yes | ** | -1649 to -288.0 |
|  | Ss261 vs Ss265 | Yes | *** | -1717 to -356.6 |

* *P* <0.05; ** *P* <0.01; *** *P* <0.001; ns non-significant.
