# Supplementary material for: Exploring virulence and immunogenicity in the emerging pathogen Sporothrix brasiliensis
Source: PLoS Negl Trop Dis. 2017 Aug 30;11(8):e0005903. doi: 10.1371/journal.pntd.0005903 (PMC5595342; doi:10.1371/journal.pntd.0005903)
Supplement: S2 Table — The percent weight loss was determined by measuring each animal’s weight every week post-inoculation (up to 16 weeks) and comparing it to the animal’s weight on the day of inoculation. Data were analyzed by paired t-test. P≤0.05 was considered significant. All analyses were performed using GraphPad Prism version 6 for Windows. (DOCX) [file pntd.0005903.s002.docx]

**S2 Table.** Statistical analysis of the weight loss assay. The percent weight loss was determined by measuring each animal’s weight every week post-inoculation (up to 16 weeks) and comparing it to the animal’s weight on the day of inoculation. Data were analyzed by paired t-test. *P*≤0.05 was considered significant. All analyses were performed using GraphPad Prism version 6 for Windows.

| **Comparison** | **Significant?**  ***P* < 0.05?** | ***P* value summary** | ***P* value** | **95% CI of diff** | **Development** |
| --- | --- | --- | --- | --- | --- |
| Control vs. Ss174 | Yes | **** | < 0.0001 | 23.56 to 48.71 | Impaired development |
| Control vs. Ss226 | Yes | *** | 0.0006 | 20.69 to 51.41 | Impaired development |
| Control vs. Ss66 | Yes | **** | < 0.0001 | 32.04 to 52.47 | Impaired development |
| Control vs. Ss99 | Yes | **** | < 0.0001 | 29.05 to 52.11 | Impaired development |
| Control vs. Ss06 | Yes | *** | 0.0001 | 12.65 to 30.93 | Moderate development |
| Control vs. Ss34 | Yes | **** | < 0.0001 | 12.65 to 22.51 | Moderate development |
| Control vs. Ss54 | Yes | **** | < 0.0001 | 24.19 to 37.98 | Moderate development |
| Control vs. Ss67 | Yes | **** | < 0.0001 | 13.68 to 26.53 | Moderate development |
| Control vs. Ss104 | Yes | **** | < 0.0001 | 14.07 to 29.04 | Moderate development |
| Control vs. Ss261 | Yes | **** | < 0.0001 | 14.86 to 27.72 | Moderate development |
| Control vs. Ss265 | Yes | **** | < 0.0001 | 20.52 to 35.17 | Moderate development |
| Control vs. Ss252 | Yes | **** | < 0.0001 | 13.32 to 28.99 | Moderate development |
| Control vs. Ss39 | No | ns | 0.0519 | -0.03342 to 7.296 | Normal development |
| Control vs. Ss126 | Yes | ** | 0.0063 | 3.038 to 15.44 | Normal development |

Control = PBS group; * *P* <0.05; ** *P* <0.01; *** *P* <0.001; *** *P* <0.0001; ns non-significant.
